# AID-induced remodeling of immunoglobulin genes and B cell fate

**DOI:** 10.18632/oncotarget.1546

**Published:** 2013-12-14

**Authors:** Brice Laffleur, Nicolas Denis-Lagache, Sophie Péron, Christophe Sirac, Jeanne Moreau, Michel Cogné

**Affiliations:** ^1^ Limoges University, Limoges France; ^2^ Centre National de la Recherche Scientifique; ^3^ Institut Universitaire de France, Limoges, France

**Keywords:** AID, B cell fate, BCR, immunoglobulin genes

## Abstract

Survival and phenotype of normal and malignant B lymphocytes are critically dependent on constitutive signals by the B cell receptor (BCR) for antigen. In addition, either antigen ligation of the BCR or various mitogenic stimuli result in B cell activation and induction of activation-induced deaminase (AID). AID activity can in turn mediate somatic hypermutation (SHM) of immunoglobulin (Ig) V regions and also deeply remodel the Ig heavy chain locus through class switch recombination (CSR) or locus suicide recombination (LSR). In addition to changes linked to affinity for antigen, modifying the class/isotype (i.e. the structure and function) of the BCR or suddenly deleting BCR expression also modulates the fate of antigen-experienced B cells.

## 1 AID FUNCTION AND SITES OF ACTION

Activation-induced deaminase (AID), an ancestral AID/APOBEC family member, deaminates DNA cytidines into uridines within immunoglobulin (Ig) variable (V) regions in all vertebrate species carrying B cells, thus supporting their Ag-driven diversification through gene conversion (GCV) and/or somatic hypermutation (SHM) [1]. It also diversifies expression of Ig heavy chain (IgH) constant (CH) regions in frogs, birds and mammals, who have developed class switch recombination (CSR) of CH genes.

AID was first identified as specifically expressed during the antigen-driven B cell maturation that mostly occurs in germinal centers (GC) of peripheral lymphoid organs [2]. It is mandatory for SHM and CSR [3] while its defect in patients results in hyper-IgM immune deficiency [4]. Its random mutagenic activity alters V domain complementarity determining regions, and thus modulates BCR (and later on antibody) binding affinity in a selection process where SHM is coordinated with cell competition for optimal intra-GC interactions with antigen-presenting cells [5]. In some mammals, especially in cattle, AID-mediated SHM can also begin in fetal gut associated lymphoid tissues prior to any contact with exogenous antigens [6].

Biochemically, G:U mismatches created through AID deamination can be processed in several ways, preferentially leading to mutations rather than repair within Ig genes. In « phase 1 » mutations, direct replication across a G:U mismatch can generate transitions from G:C to A:T base pairs. Base excision repair (BER) and uracil removal by uracil N-glycosylase (UNG) instead generate abasic sites, which subsequently undergo DNA nicking by apurinic/apyrimidinic endonuclease, and are repaired during replication by error-prone DNA polymerases as both transitions and transversions. G:U mismatches can also be processed by the mismatch repair (MMR) pathway involving MSH2/MSH6, with associated error-prone DNA polymerases and then result in patches of « phase 2 » mutations at both G:C and (preferentially) A:T base pairs around targeted cytosines.

Primary regulation of AID activity in B cells relies on its strictly controlled tissue-specific and stage-specific expression upon cell activation, due to control of the level of AID transcripts by both ubiquitous and lymphoid-specific transcription factors (Pax-5, STAT6, SP1, C/EBP) and miRNAs (miR155 and miR181b). This ensures high AID expression only in activated B cells with appropriate signals, as occurring within GCs upon interaction with follicular dendritic cells and T follicular helper cells. In addition, AID can appear at low levels in some bone marrow developing B cells upon stimulation of toll like receptors (TLR) [7, 8].

**Figure 1:**
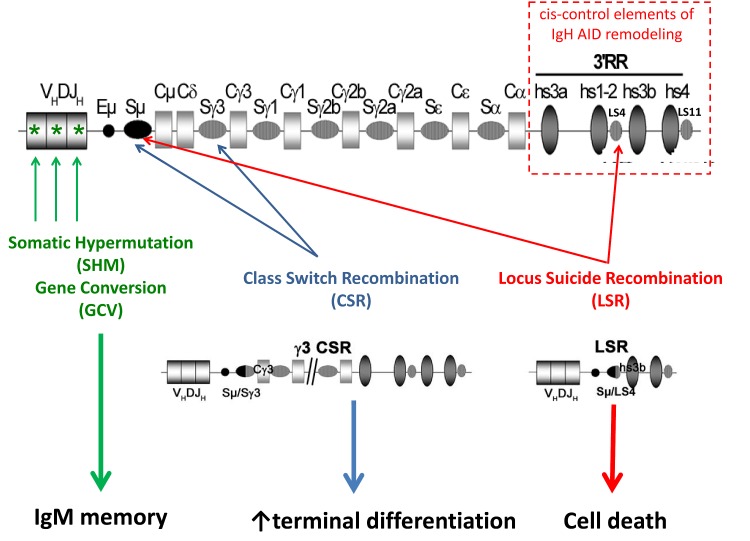
AID targeting of the IgH locus Upon B cell activation, induced AID expression remodels Ig gene V regions through SHM or eventually gene conversion (GCV), generating B cell receptors of improved affinity for antigen. B cells, in parallel or later, diversify the BCR class through class switch recombination (CSR). Locus suicide recombination (LSR) eventually joins the switch μ region (Sμ) with one of the like-switch (LS) repetitive regions located within the 3' regulatory region (3'RR), then deletes all IgH constant genes and switches off BCR expression, thus leading to B cell death.

AID requires transcription of target regions and also preferentially deaminates cytosine into uracil within WRC motifs (W = A/T, R = A/G) [9]. Besides potential constraints concerning the “accessibility” of target DNA, another major link between AID targeting and transcription is that AID loading onto Ig genes requires physical interaction with stalled RNAPII and bound Spt5 that occurs immediately downstream from transcription start sites [10]. The RNAPII associated polymerase associated factor (PAF) complex also helps recruit AID [11]. CH regions are protected from AID attack due to the absence of RNAPII pausing. Switch (S)-region transcription before AID recruitment is under the control of cytokine-dependent germline promoters preceding CH regions and a series of B cell activation-dependent transcriptional enhancers located in the 3' regulatory region (3'RR) of the IgH locus [12–15]. While AID generates mutations in V regions, it initiates DNA breaks (DSBs) in S regions, thereby promoting large deletions [16, 17]. DSBs activate the ubiquitous DNA damage response, which is then resolved through classical (C-) or alternative non-homologous end joining (A-NHEJ). Recruitment of 53BP1 and Rif1 [18] to broken DNA ends (and subsequent formation of γH2AX foci) is required for protection of DNA ends from resection before repair and ligation by C-NHEJ rather than A-NHEJ [19, 20]. AID recruitment to both V and S regions (and S-S region synapses, likely favored by IgH locus DNA loops) requires IgH 3'RR enhancer activity elements [13] [15] [14] [21] [22]. Multiple 3'RR genetic alterations affected transcription of AID targeted regions [12–15]. However, transcription was often partially reduced while being associated with complete CSR and/or SHM blockades. In addition to boosting transcription, the 3'RR thus likely promotes AID activity through epigenetic changes of targets, or by attracting and recruiting AID and/or AID partners. Figure 1 resumes the different targets of AID in the IgH locus.

## 2 CSR AND BCR CLASS-SPECIFIC CONTROL OF B CELL FATE

### 2.1 Structure and function of the B Cell Receptor

The B cell receptor is composed of a membrane-bound immunoglobulin (mIg) associated with the disulfide-linked heterodimer transducing module CD79A / CD79B (Igɑ / Igß). The mIg is composed of two heavy (H) and two light (L) chains, each including a variable (V) domain encoded after RAG-mediated recombination of Ig gene segments (for review [23]). Within mIgs, constant (C) regions carry additional transmembrane and cytoplasmic domains which support interactions with Igɑ/ß and then accessory receptors and downstream intracellular signaling cascades including the src kinases Syk, Lyn and adaptor proteins such as BLNK for interactions with PLCγ2, PI3K, calcium and glucose transporters. IgH C domains confer their class specific effector functions to the corresponding secreted Ig.

### 2.2 IgM/IgD BCR

**Figure 2:**
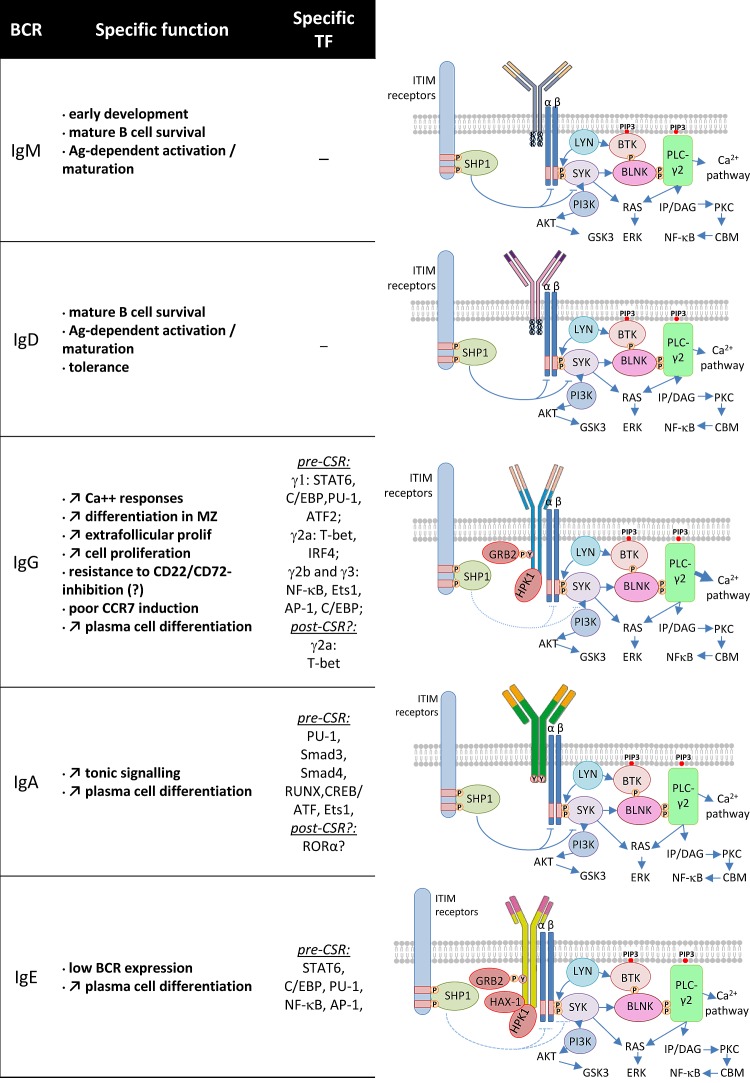
BCR class and signaling cascades Some specific functions of the various BCR classes are indicated (left). Specific transcription factors expressed before CSR to a given class (and eventually after CSR) are mentioned (middle). Each BCR class is represented as a membrane Ig associated with the Igɑ/ß signaling modules (right). The three amino acids (KVK) of the IgM and IgD BCR and longer intra-cytoplasmic tails of other classes are shown. The main signaling proteins and pathways that are common or most likely specific to these BCR classes are represented (right).

While all mIg associate with the Igɑ/ß complex to constitute the BCR, signaling from this complex has mostly been studied for IgM [24][25]. The membrane μ HC has multiple roles by first providing differentiation signals during early development, survival signals in resting B cells and activation signals during peripheral antigen-dependent maturation (Figure 2). IgM and IgD have an identical intracellular positively charged short tail (including residues KVK). Replacement of μ by δ HC expression resulted in a modest phenotype and suggested that IgM and IgD can signal in a roughly similar manner [26]. This replacement completely deleted both mIgM expression and secretion of soluble IgM. The main feature of the resulting phenotype was delayed affinity maturation of immune humoral responses, i.e. a phenotype similar to mice simply lacking the secreted form of IgM [26][27]. It was also shown later that, despite their roughly normal peripheral B cell compartments, mice in which μ HC was replaced by δ HC expression had a partial blockage of pro-B to pre-B transition due to lack of autonomous pre-BCR signaling normally provided by a glycosylation site within the μ chain CH1 domain [28].

IgD-deficient mice have also been generated through gene targeting and only showed slightly reduced B-cell spleen and lymph node compartments with higher mIgM expression than wt B cells. Early and late B cell differentiation, GC formation and humoral responses to immunization were unaffected [29]. However, a later study showed autoantibodies accumulating in IgD-deficient/lpr mice, suggesting that mIgD plays a role in B cell tolerance and limits auto-reactive B cell differentiation into plasma cells [30].

### 2.3 mIgG BCR

There are clear qualitative differences in BCR signaling and responses to antigen that are conferred by IgG vs IgM HC tails [31] (Figure 2). IgG carry longer and highly conserved cytoplasmic tails. Deletion or replacement of the IgG tail with an IgM tail in mice resulted, for unexplained reasons, in lower levels of membrane expression and also strikingly disturbed IgG1 memory, showing that the IgG tail is necessary for high-titer IgG secretion and IgG1-switched memory cell survival [32]. In contrast, studies in transgenic mice carrying IgM, IgG or IgM/G (IgM with an IgG tail) BCRs of identical antigen specificity for hen-egg lysozyme, showed that the IgG tail increased production of plasma cells during extra-follicular T cell−dependent antibody reactions [33]. It was also observed in B cell transfectants that signaling through the mIgG BCR, but not through mIgM nor mIgD, was resistant to CD22-mediated signal inhibition [34]. However, this CD22-independence was not confirmed by other detailed studies in primary cells from transgenic or knock-in mice [31][35]. Rather, it was then suggested that IgG enhancement of intracellular calcium responses did not reflect independence from CD22 inhibition or changes in tyrosine phosphorylation, ERK signaling, or global gene induction, but increased differentiation into marginal zone cells, extrafollicular proliferation, and plasma cell differentiation. Despite increased Ca2+ responses, several BCR ligation response genes showed lower induction by the IgG tail, in agreement with the observation that marginal zone B cell differentiation is favored by conditions that decrease BCR signaling [36]. This includes poor CCR7 induction by antigen ligation of the IgG BCR, while before entry into follicles, CCR7 is known to attract B cells to CCL19/CCL-21-rich T-zones rather than towards the marginal zone.

Premature expression of IgG rather than IgM in B cell progenitors also resulted in abnormal pre-BCR signaling and partial blockage of pro-B to pre-B cell transition, emphasizing that mIgM is optimal for early B cell maturation. In contrast, maintenance of mIgG+ cells might be less dependent on Igα/β than mIgM+ cells [35]. These observations led Goodnow's group to propose a “less-is-more” hypothesis where decreased induction of some BCR response genes might increase plasma cell formation, and to globally conclude that signaling differences between IgG and IgM BCR classes were qualitative rather than quantitative [31].

In comparison to IgM, another specific signaling feature of mIgG (shared with mIgE), is recruitment of the adaptor Grb2, which can bind to a tyrosine of the mIgG intracellular tail upon phosphorylation, and then modulate signaling and increase cell proliferation [37]. Interestingly, Grb2 is a modulator of cell signaling by tyrosine kinase receptors, with the dual function, in some instances, to decrease basal signaling while increasing signals emanating from receptor ligation [38].

Another feature of the mIgG1 cytoplasmic tail was demonstrated: enhancing early BCR oligomerization that immediately follows ligation of membrane bound antigen leading to increased Syk recruitment and calcium mobilization [39]. Importantly, a recent study has shown that the strong tendency of mIgG1 cells to differentiate into plasma cells is restricted to those Ag-experienced cells and then translated into Bach2 repression, thus clearly involving more than just mIgG intracellular tail structure [40].

### 2.4 mIgA BCR

Few studies have explored a potential role for mIgA in conferring specific properties to memory mIgA+ B cells in comparison to naive mIgM+ cells (Figure 2). mIgA cross-linking raises intracellular calcium concentrations so that mIgA+ B cells residing in mucosa associated lymphoid tissues (MALT) can mediate IgA responses to local immunization [41][42]. As for IgG, it was also shown by targeted deletion in the mouse, that the Cα membrane anchoring region allowing mIgA expression, is necessary for differentiation of IgA secreting cells in vivo [43]. The μMT mutation removing Cμ membrane exons and IgM/IgD expression did not result in a complete B cell defect but resulted in accumulation of IgA+ plasma cells in MALT, also suggesting that B cell progenitors can undergo CSR to mIgA, survive under expression of this BCR and then differentiate into ASCs [44]. That early B cell development can be ensured by expression of membrane α HC has been shown in the α1KI model where the Sμ region of the mouse IgH locus was replaced with an Ig HC ɑ1 gene [45].

In α1KI mice, a partial defect in pre-B cells was, however, noticed, indicating that the α-class pre-BCR was less efficient than μ-class pre-BCR for signaling completion of V(D)J rearrangement upon assembly of a functional pre-BCR or BCR complex. This was reminiscent of data in mice with premature expression of membrane γ HC instead of μ/δ [35].

Despite this developmental defect, B cells accumulated in lymphoid organs of ɑ1KI mice including spleen follicles, marginal zone, lymph nodes, Peyer's patches and the peritoneal B1 compartment. Splenic marginal zone and follicular B cell numbers were affected in the same proportion, in contrast to the increased marginal zone differentiation reported in mice expressing an IgG BCR [31, 35]. As mentioned in a previous study [32], BCR density appeared lower in ɑ1KI than in wt mice, despite normal association of mIgA1 with Igɑ/ß. Another feature of mIgA-driven B cell differentiation in ɑ1KI mice was the abundance of plasma cells, affecting to the same extent long-lived (accumulated) and short-lived (recently differentiated) plasma cells. Rather than just compensatory plasma cell accumulation in response to B cell lymphopenia, the α1KI intrinsic commitment to plasma cell differentiation was also manifested in vitro by higher differentiation of B cells activated by BCR cross-linking or mitogens into CD138+ cells.

Interestingly, normal human blood mIgA+ cells also include a high proportion of CD38 expressing cells, a marker of engagement in plasma cell differentiation [46]. It should also be noticed that the IgA BCR has been shown to be sensitive to CD22 inhibition as for IgM [47]. In addition, a recent study demonstrated a specific transcriptional program for mIgA+ memory cells, notably depending upon high RORɑ expression, while mIgG2a+ cells were intrinsically dependent upon high expression of T-bet [48].

### 2.5 mIgE BCR

Secreted IgE is the least prevalent Ig class in blood, with only nanograms per milliliter compared to micrograms for IgD and milligrams for other classes in healthy populations. Maintaining low IgE levels is controlled by stringent mechanisms in order to restrict the very potent effects and side-effects of IgE immune responses. The IgE molecule is evolutionarily conserved and present in all mammalian species studied. In contrast to its active role in immune responses against parasites, IgE can also be deleterious and mediate atopic diseases of various intensities (eventually lethal in the case of anaphylactic shock).

Production of the double-sworded IgE antibody thus requires tight regulation, for which multiple mechanisms might be at work. IgE expression was first postulated to be restrained at the CSR level. Whereas IgG1 and IgE class switching can be both induced by IL4 in mice, in vitro experiments most often show lower switching frequency to IgE than to IgG1 [49]. Preferential CSR to Cγ1 might thus reflect intrinsic differences between the 10kb-long Sγ1 and the much shorter SƐ region. Furthermore, IgE class switching is often a sequential process with an IgG1 intermediate [50]. Interestingly, direct CSR to IgE (without prior IgG1 CSR) occurs preferentially in immature B cells, and this feature may reflect epigenetic marks modulating accessibility of the IgH locus during B cell differentiation [51].

Beyond IgE CSR, it is clear that expression of mIgE BCR is mandatory for further differentiation into IgE secreting plasma cells (Figure 2). Mice deleted for IgE membrane exons have reduced (by more than 95 percent) serum IgE levels and fail to develop antigen specific IgE responses [52]. To a milder extent, replacement of the IgE tail with the much shorter IgM tail also decreased (~50%) IgE secretion [52]. It was also shown that treatment of mice with an antibody specifically targeting a mIgE-specific epitope on mIgE+ cells efficiently inhibited secreted IgE production [53][54], further suggesting that mIgE+ B cells are a necessary step for differentiation of IgE secreting plasma cells.

In this regard, a structural feature of the Cε gene was postulated to restrain the production of membrane-type Cε transcripts which drive mIgE expression. Whereas all documented Ig genes have classical polyA sites (AATAAA) after both their secretory (CH3 or CH4) and membrane (M1/M2) exons, human and mouse Cε genes carry an atypical polyA site downstream from the M2 exon [55]. By comparing the sequence of CƐ genes in the IMGT data base (http://www.imgt.org), we observed that a canonical AATAAA sequence downstream from CƐ M2 was absent in all published mammalian IgH loci. This conserved suboptimal polyA site may down-regulate the ratio of membrane- vs secreted-form Cε mRNA transcripts during alternative splicing of murine (and human) Cε primary transcripts [56] and thus limit mIgE BCR expression. In an utmost form, CƐ gene membrane exons are lacking in one lineage of primates, the tarsiers [55].

Despite these limitations, mIgE expression can be characterized in vitro in human or mouse stimulated B cells and has also been characterized in a B lymphoma cell line [57]. Exploring mIgE expression requires rigorous staining protocols eliminating passive binding of soluble IgE to FcεRII receptors (whose expression is strongly induced in the presence of IL4) [58].

While present in a single (short) form in most mammalian species, mIgE is additionally present in humans and apes as a long isoform generated through alternate splicing of the M1 exon, and including either a long (66 amino acids) or a short (14 amino acids) extracellular membrane proximal domain (EMPD) between CH4 and the transmembrane domain. Both human mIgE isoforms have been expressed as functional BCRs in transfected B cells, showing that the short isoform was more efficiently transported to the cell surface and that its ligation resulted in growth inhibition of the WEHI immature B cell line, similar to mIgM BCR [59]. The EMPD domain was shown to be necessary for B cell activation through mIgE ligation in transfectants from the mature B cell line A20, while its deletion yielded a form of mIgE with proapoptotic activity upon ligation [60].

Another particularity of the IgE BCR is the intracellular tail structure, and phage display experiments identified 2 proteins interacting with the IgE tail. HS1-associated protein X-1 (HAX-1) notably seems to be associated with the IgE specific YANIL motif (where Y is a potentially phosphorylated tyrosine) of the intra-cellular tail and may influence BCR mediated Ag-internalization and cognate Ag-presentation to T cells [61]. Hematopoietic progenitor kinase 1 (HPK1) was also shown to interact with mIgE and mIgG (but not mIgM) tails and could differentially affect signaling from these receptors [62]. IgE committed B cells were also shown to be exquisitely sensitive to inhibition through ligation of LXR receptors, potentially due to increased CD23 expression and in turn down-regulating IgE production [63].

In order to study IgE responses in vivo, different mouse models have been developed and characterized. Xiong et al used T and B monoclonal mice (with anti-chicken ovalbumin (OVA) T cell receptor transgenes and anti-influenza hemagglutinin (HA) knock-in B cell receptor genes on a RAG1-deficient background), immunized with OVA-HA and OVA-PEP1 (a variant of HA) and a classical model of helminth infection in wt mice. They showed that IgE+ cells were found outside the GC (in contrast to IgG1 cells) and displayed a plasma cell transcriptional program. They also showed that IgEs accumulate hypermutation and affinity selection (and are able to bind the PEP1 Ag) and proposed a sequential maturation program: a pre-IgE phase with SHM and affinity maturation in IgG1+ cells and a post-IgE-switching phase where IgE+ rapidly differentiate into plasma cells [64]. In another study this team used the same T/B monoclonal model and immunized wt or hMT mice (which are deficient for IgG1 populations) with NP19KLH Ag. They demonstrated that high affinity IgE Abs responsible for anaphylactic reactions were generated through the sequential μ/γ1/Ɛ CSR pathway (and were absent in hMT mice), with IgG1 intermediate cells supporting SHM and affinity selection. In contrast, low affinity IgE producing cells were generated through direct μ/Ɛ CSR, were less mutated, and may play a beneficial role during anaphylaxis, by competing with high affinity Ab for FcƐR1 binding [65].

Two transgenic models were created to study rare IgE+ cells in vivo. The first model used a GFP reporter system by inserting a bi-cistronic GFP reporter gene downstream from the mouse IgE M2 exon. The long human EMPD domain was introduced upstream to the IgE M1 exon, resulting in a chimeric IgE BCR with an additional EMPD domain (allowing specific detection of these cells but denaturing the natural architecture of this receptor). This model was subjected to helminth infection by N. brasiliensis and TNP-OVA immunization. In contrast to previous models, this strategy demonstrated IgE+ cells in GC and suggested the existence of IgE+ memory and plasma cells [66]. In another model, the T2A strategy tagged IgE+ cells by linking the Cε M2 exon with Venus (a derivative of yellow fluorescent protein) and used a viral 2A sequence enabling expression of 2 proteins from a single ORF. Translation of Venus was thus linked to translation of a nearly normal mIgE (except for an additional 17 amino acid peptide). These mice and wt mice were immunized with NP-KLH and infected with N. brasiliensis. This model suggested that IgE+ cells can differentiate into GC B cells during primary immune responses and confirmed a strong propensity of these cells to differentiate rapidly into plasma cells which were mostly short-lived [67]. In agreement with this conclusion, another model where membrane IgE exons were replaced by membrane IgG1 exons suggested that IgE+ plasma cells have an intrinsically lower chance than IgG1+ cells to migrate towards the chemokine CXCL12 and thus to contribute to the long-lived plasma cell pool [68].

## 3 LSR ELIMINATION OF B CELLS

B cell fate can also be more dramatically modified by AID after CSR-like events resulting in complete deletion of the IgH CH gene cluster and thus inducing B cell death through locus suicide recombination (LSR) (Figure 1) [16].

**Figure 3:**
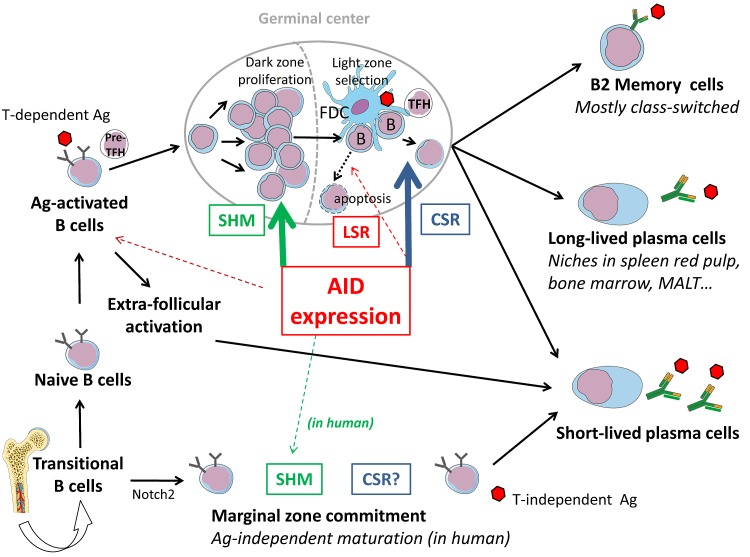
AID-modulation of B cell fate in the context of lymphoid tissues After V(D)J recombination and IgM expression, recently emerged B cells circulate as transitional cells and can be committed to various compartments depending upon BCR signalling (Notch2 expression and weak BCR tonic signaling commit B cells to a marginal zone fate). After maturation into IgM+, IgD+ cells, naïve B cells encounter T-dependent Ag and undergo cognate interactions with pre-TFH cells. B cells then initially activate in extra-follicular foci and differentiate into short-lived plasma cells, or participate with TFH in the formation of GCs where their BCRs will be deeply remodeled by SHM (mostly in the proliferating dark zone) or CSR (mostly in the GC light zone), or eventually LSR. The GC reaction will yield both memory cells and plasma cells, some being long-lived and surviving after their migration to several protective niches (spleen red pulp, bone marrow, MALT…).

During AID-mediated competition that occurs among B cells within germinal centers, a few with high affinity are selected (Figure 3). Besides these winners, many cells are losers or undesired responders deserving elimination. Although some unfavorable mutations of V regions can promote apoptosis [69], abundant survival signals from the GC microenvironment might also activate or maintain bystander cells with useless or eventually harmful BCRs (the latter having, for example, acquired specificity for self or environmental antigens after random remodeling of V sequences). Since class-switched antibodies are potent actors of auto-immunity and/or hypersensitivity, means for restricting CSR and reentry of class-switched cells into SHM would help ensure the specificity of immune responses. Yet, how the post-GC repertoire is controlled remains poorly understood.

The 3'RR contains several enhancers with strong B-lineage specificity [70,71] undergoing chromatin remodeling upon B cell activation and controlling V region SHM as well as germline transcription and CSR to most C genes [72–83]. In all mammalian species studied, the 3'RR also features inverted repeats and stretches of repetitive DNA with structures similar to that of S regions [84–87], showing multiple 5bp repeats eventually arranged within higher order (49bp) repeats. Such “like S” stretches (LS regions) flank both sides of the 3'RR and are also interspersed with 3' enhancers. Similarly to the germline transcription of S regions preceding CSR, the 3'RR is transcribed by RNA polymerase II in activated B cells [88].

3'RR elements regulate AID-mediated SHM and CSR by physically interacting with AID-targeted IgH promoters [70, 74, 79, 80, 89]. Since 3'RR enhancer elements and LS regions are themselves transcribed, it was logically found that they also undergo SHM and DNA breaks. AID-initiated junctions then occur in activated B cells between Sμ and 3'RR LS regions and delete the complete C gene cluster, thereby eliminating BCR expression (or Ig secretion for cells engaged in plasma cell differentiation). Given that BCR expression is mandatory for B cell survival, this process termed “locus suicide recombination” (LSR) necessarily leads to B cell death.

How the CSR/LSR balance is finely regulated in vivo remains to be determined. Beyond IgH locus accessibility, intra-GC interactions govern the selection of high affinity class-switched cells. In this context, LSR might contribute to B cell homeostasis and lead to death of suboptimally stimulated cells, while optimal stimulation would promote CSR and survival of selected antigen-specific B cell clones. LSR could then help shape the peripheral B cell repertoire and ensure BCR antigen specificity.

## 4 DEVELOPMENT AND FEATURES OF MEMORY B CELLS

B cell responses to T-dependent antigens involve germinal center reactions featuring B cell proliferation and expression of AID for SHM and CSR in parallel interactions with follicular dendritic cells and T follicular helper cells that will help select those B cells with the highest affinity for Ag. GC reactions peak after 7 days and wane after a few weeks, once having generated long-lived plasma cells and memory B cells with an affinity matured BCR. Besides intra-GC competition between B cells for limited antigen, soluble antibody feedback also ensures inter-GC communication concerning access to antigen [90]. Secreted antibodies were thus shown to inhibit or even terminate GC proliferation with an efficiency directly correlated with their affinity for antigen [90]. Interestingly in the absence of T cell help, soluble antigen might also inhibit GC development [91].

Regarding the prolonged maintenance (eventually lifelong) of GC-born memory B cells, it was shown that Ag-persistence within lymphoid organs is unnecessary [92]. Rather, further reintroduction of Ag into the organism will trigger memory cell differentiation into plasma cells, with or without parallel formation of new GC [93]. A recent study by Reynaud and colleagues followed Ag-specific memory B cells for up to 1 year in mice and showed that while GCs waned a few weeks after immunization by a soluble antigen, they persisted up to 10 months after immunization with a particulate Ag [94]. Several types of memory cells were demonstrated in the latter model: either IgM+IgD+, or IgM-only, or class-switched (and then mostly mIgG1+). Ag-experienced cells were located in the spleen T cell zone, red pulp, and follicles, with very few in the marginal zone. Upon transfer into pre-immunized recipient animals, the mIgM+ subset gave rise to centroblasts, part of them switching to IgG1, and to a small proportion of Ag-specific IgM+ plasma cells. The mIgG1 subset yielded Ag-specific IgG1 plasma cells and maintenance of a mIgG1+ memory pool. While the average number of mutations was stable in persisting memory B cells, it was increased upon differentiation of mIgM+ memory cells into centroblasts, showing that such cells can reenter new cycles of AID-mediated affinity maturation.

In another model, two layers of Ag-specific B memory cells were demonstrated, either mIgM+ or having undergone class switching (swIg+ memory cells). The mIgM+ memory cells were very stable and long-lived, able to form GC when transferred into a naïve animal but inhibited upon the presence of soluble Ag-specific Ig (then inhibiting GC formation). The swIg+ memory cells persisted for a shorter time, and mostly differentiated into plasmablasts upon activation but kept the ability to be activated even in the presence of preexisting antibodies. This led to the proposition that memory 1) initially relies on swIg+ cells, able to be rapidly activated and yield antibodies even in the presence of preexisting serum antibodies, and 2) later relies on long-lived mIgM+ memory cells, able to form new GC once initial antibodies have vanished [93]. If this model can be generalized, “early” memory responses would classically overwhelmingly involve class-switched antibodies, while “late” memory responses would be expected to recapitulate the kinetics of the primary response, first with IgM (albeit with low affinity) then followed with high affinity class-switched antibodies. Interestingly, it was recently shown in a model of bacterial infection that in vivo depletion of the mIgM memory B cells abrogated the IgG recall responses to specific Ag challenge, demonstrating that mIgM cells were then required for humoral memory, and underwent CSR and plasma cell differentiation upon Ag re-challenge [95].

Upstream of GC reactions, some specific functions have been reported during immune responses for B cells with low affinity BCRs. For example, mouse B1 cells show polyreactivity and bind multiple exogenous antigens or auto-antigens with low affinity, being responsible for generation of natural antibodies that preexist prior to any antigenic challenge and play an innate function. Some tissue B cells binding antigens with low affinity (notably in lung, with intra-nasally administered antigens) have also been demonstrated to shuttle and carry antigens towards spleen follicles, then initiating GC formation by resident B cells with higher affinities [96]. BCR independent Ag-binding by B cells was also reported for marginal zone B cells recognizing particulate antigens within immune complexes through complement or Fc receptors, which were also postulated to carry antigens towards FDCs within follicles [97]. Human and mouse MZ B cells might somehow differ although they both respond to T-independent antigens. While mouse MZ B cells have mostly unmutated BCRs and do not recirculate, human IgM+IgD+CD21highCD27+ B cells recirculate in blood and carry mutated Ig and Bcl6 genes. Their V(D)J repertoire is very large without clonal restrictions and hallmarks of antigen-induced activation, similar to naive cells and in contrast to GC mIgM+ cells, suggesting that MZ B cells provide a GC-independent IgM memory layer [98]. MZ B cells likely differentiate through an innate extracellular TLR-dependent pathway allowing AID induction outside GC, as demonstrated in both mouse and humans for immature B cells then undergoing CSR and SHM [99][7]. A helper role for neutrophils in the induction of MZ B cell CSR, SHM and plasma cell differentiation was also demonstrated during T-independent responses to bacterial antigens [100].

## 5 LYMPHOMAGENESIS AND BCR SIGNALS

AID also induces mutations and breaks in numerous non-Ig genes, many of which (but not all) are transcribed in B cells, and many of which overlap with translocation or recombination sites found in B cell lymphomas or correspond to repetitive DNA sequences. [101,102]. Studies of B cell malignancies since the early 1980s also identified a number of translocations linking oncogenes such as c-MYC, c-MAF, CYCLIN D1, and BCL2 to breakpoints located within Ig loci. Some of these events were recurrently identified as the clonal driving forces of lymphomagenesis. This pointed to a major role of errors in the processes of antibody gene diversification in tumorigenesis. Recently, it was also shown that breakpoints located outside Ig loci constitute frequent side targets of AID attacks in activated B cells. It is thus now clear that in B cell malignancies as in normal B cells, AID-induced mutations accumulate on multiple loci including those of Ig (with the higher level of error-prone repair after AID deamination), BCL6 and a large list of oncogenes and tumor suppressors.[101] Precise characterization of translocation breakpoints and development of whole genome sequencing showed that mutations and recombinations detected in activated B cells and in tumors with mature B cell phenotypes all carry hallmarks of WRC sequence-specific AID on-target and off-target attacks.[103,104] In addition, more than half of translocations seen in immature B cell malignancies also show breakpoints at sites of AID deamination corresponding to mCG dinucleotides and might imply the combined action of AID, RAG and ARTEMIS. [105,106] However, this issue can still be debated and it is noteworthy that lymphoma breakpoints targeting CG dinucleotides were also recently identified by A. Nussenzweig and colleagues, as early replicating fragile sites susceptible to AID-independent genomic instability [107].

Besides the role of translocations, it is important to note that BCR signals most likely play a crucial role in lymphomagenesis. These signals might vary with SHM of V regions, which probably modulate interactions of malignant cells with various endogenous ligands. Indeed, N-glycosylation sites carrying high-mannose glycans and created by SHM within V domains are a frequent feature of follicular lymphoma cells, and may promote B cell interactions with lectins such as DC-SIGN [108]. The BCR Ig class might also somehow control the phenotype of B cell malignant proliferations. Indeed, it is well known that plasmacytic lymphomas overwhelmingly express mIgM (in striking contrast with myelomas, where the secreted Ig is always a non-IgM class). In diffuse large B cell lymphomas, it was recently shown that ABC cases mostly express mIgM, while GC cases overwhelmingly express IgG or IgA [109].

Autonomous BCR signaling in chronic lymphocytic leukemia cases was also recently demonstrated, but appeared independent from the mutated or unmutated BCR status [110].

Finally a number of AID-mediated mutations affecting proteins of BCR- or TLR-signaling cascades and resulting in constitutive B cell activation have been observed in many different types of lymphoproliferation [111,112].

## 6 CONCLUSIONS

Early B cell differentiation is optimally driven through expression of germinally encoded and diversified IgM molecules. This generates transitional B cells that are affected to the various mature B cell compartments and then express variable levels of IgM and IgD. Such cells can then undergo either Ag-dependent (within B cell follicles) or Ag-independent (for splenic marginal zone B cells), AID remodeling of their Ig V regions through SHM or GCV. Cell survival, fate and phenotype are all highly dependent at that time on constitutive signals provided by the BCR. Either antigen ligation of the BCR and/or stimulation by various TLR ligands can additionally result in B cell activation and rapid AID induction. By improving BCR affinity for antigen by SHM, AID will help select follicular cells having the strongest interactions with antigen exposed at the surface of FDC along the process of SHM, as winners of the competition for activation and survival signals provided by FDC and TFH cells within the GC. In parallel, these interactions further stimulate expression and accessibility of the IgH locus then resulting in CSR for expression of a new BCR with conserved Ag-specificity but new signaling functions. Finally in some instances, another dramatic IgH remodeling event implying complete deletion of the IgH locus C region through a “sterile CSR” (also defined as ”locus suicide recombination” or LSR), can occur and result in loss of BCR expression and B cell death.
